# Metagenomic investigation of vestimentiferan tubeworm endosymbionts from Mid-Cayman Rise reveals new insights into metabolism and diversity

**DOI:** 10.1186/s40168-018-0411-x

**Published:** 2018-01-27

**Authors:** Julie Reveillaud, Rika Anderson, Sintra Reves-Sohn, Colleen Cavanaugh, Julie A. Huber

**Affiliations:** 1 ASTRE, INRA, CIRAD, University of Montpellier, Montpellier, France; 2 Josephine Bay Paul Center, Marine Biological Laboratory, Woods Hole, MA USA; 3 Department of Biology, Carleton College, Northfield, MN USA; 4 Department of Organismic and Evolutionary Biology, Harvard University, Cambridge, MA USA; 5 Present Address: Marine Chemistry and Geochemistry, Woods Hole Oceanographic Institution, Woods Hole, MA USA

## Abstract

**Background:**

The microbial endosymbionts of two species of vestimentiferan tubeworms (*Escarpia* sp. and *Lamellibrachia* sp.2) collected from an area of low-temperature hydrothermal diffuse vent flow at the Mid-Cayman Rise (MCR) in the Caribbean Sea were characterized using microscopy, phylogenetic analyses, and a metagenomic approach.

**Results:**

Bacteria, with a typical Gram negative cell envelope contained within membrane-bound vacuoles, were observed within the trophosome of both tubeworm species. Phylogenetic analysis of the 16S rRNA gene and ITS region suggested MCR individuals harbored highly similar endosymbionts that were > 98% identical, with the exception of two symbionts that showed a 60 bp insertion within the ITS region. All sequences from MCR endosymbionts formed a separate well-supported clade that diverged from those of symbionts of seep and vent vestimentiferans from the Pacific, Gulf of Mexico, and Mediterranean Sea. The metagenomes of the symbionts of two specimens of each tubeworm species were sequenced, and two distinct *Gammaproteobacteria* metagenome-assembled genomes (MAGs) of more than 4 Mbp assembled. An Average Nucleotide Identity (ANI) of 86.5% between these MAGs, together with distinct 16S rRNA gene and ITS sequences, indicate the presence of multiple endosymbiont phylotypes at the MCR, with one MAG shared between one *Escarpia* and two *Lamellibrachia* individuals, indicating these endosymbionts are not specific to either host species. Genes for sulfur and hydrogen oxidation, nitrate reduction (assimilatory and dissimilatory), glycolysis and the Krebs cycle, peptide, sugar, and lipid transporters, and both rTCA and CBB carbon fixation cycles were detected in the MAGs, highlighting key and shared functions with symbiont metagenomes of the vestimentiferans *Riftia*, *Tevnia*, and *Ridgeia* from the Pacific. The potential for a second hydrogen oxidation pathway (via a bidirectional hydrogenase), formate dehydrogenase, a catalase, and several additional peptide transporters were found exclusively in the MCR endosymbiont MAGs.

**Conclusions:**

The present study adds new evidence that tubeworm endosymbionts can potentially switch from autotrophic to heterotrophic metabolism, or may be mixotrophic, presumably while free-living, and also suggests their versatile metabolic potential may enable both the host and symbionts to exploit a wide range of environmental conditions. Together, the marked gene content and sequence dissimilarity at the rRNA operon and whole genome level between vent and seep symbionts suggest these newly described endosymbionts from the MCR belong to a novel tubeworm endosymbiont genera, introduced as *Candidatus* Vondammii.

**Electronic supplementary material:**

The online version of this article (10.1186/s40168-018-0411-x) contains supplementary material, which is available to authorized users.

## Background

Vestimentiferan tubeworms (phylum *Annelida*) often thrive in invertebrate communities in marine hydrothermal vent and cold seep sites. As the first animals in which chemoautotrophic symbiosis was described, this discovery opened up a whole new area of biological research [1, 2]. Mouthless and gutless, the tubeworms are dependent on chemosynthetic bacterial symbionts that provide them with organic compounds and nutrition. The worm acquires all of the substrates needed for chemosynthesis, including oxygen, sulfide, and carbon dioxide, from seep or vent fluids mixed with seawater, and delivers them to the symbiotic bacteria for sulfide oxidation and autotrophy. The energy produced provides carbon for growth and metabolism to the worms [1, 2]. Symbionts are contained in bacteriocytes, which are specialized host cells in a highly vascularized internal organ termed the trophosome [3]. Although tubeworms are exposed to a plethora of diverse microbes in their environment, they associate with only specific *Gammaproteobacteria* [4]. Each tubeworm generation is newly colonized with its symbionts from the environment, highlighting efficient mechanisms for recognition, colonization, and host–symbiont interactions [4–7].

Chemosynthetic symbionts have never been isolated into pure culture. Therefore, cultivation-independent studies are essential for understanding the geographic distribution, diversity, metabolism, and evolution of symbionts. In particular, metagenomic studies can provide key information about the metabolic capacities of chemosynthetic symbiosis. With the first tubeworm metagenome-assembled genome, the name *Candidatus* Endoriftia persephone was proposed for the Gammaproteobacterial endosymbiont of the vent-associated tubeworm *Riftia pachyptila* [8]. *Endoriftia* has identical or nearly identical 16S rRNA gene sequences to the vent endosymbionts of the sympatric vestimentiferan *Tevnia jerichonana* and of the geographically distant *Ridgeia piscesae* and *Oasisia alvinae* [5, 6, 9]*,* indicating widespread tubeworms establish symbioses with the same or closely related organisms. Recently, Klose and colleagues showed large numbers of viable symbionts are released upon host death, which could explain effective dispersal and colonization of new recruits to local and distant sites [10]. On the other hand, a comparative study between metagenome-assembled genomes of *R. pachyptila*, *T. jerichonana*, and newly obtained *Ridgeia* draft genomes shows symbiont populations are structured at Eastern Pacific spreading centers, with both geographic distance and host specificity playing important roles in the endosymbiont population structure [6].

Much less is known about the genomic content and distribution of endosymbionts of the tubeworm genera *Escarpia* and *Lamellibrachia*. Originally regarded as seep species and extensively studied in the Gulf of Mexico (GOM) and the Gulf of Guinea in West Africa [11, 12], these vestimentiferans have also been found in vent habitats in the Pacific along the Juan de Fuca Ridge at Middle Valley, Mariana Arc, Lau Basin [13], in the Mediterranean Sea [14], in Kagoshima Bay in Japan [15], and at ship wrecks and whale falls [16, 17], demonstrating a wide geographic distribution. Based on 16S rRNA gene analysis, *Escarpia* and *Lamellibrachia* symbionts from the GOM and the Mediterranean Sea share almost identical 16S phylotypes, although they cluster within a “seep” clade, distinct from the “vent” clade that includes *Riftia*, *Oasisia*, *Tevnia,* and *Ridgeia* [9, 13, 14].

Recently, the first tubeworms found in the Atlantic at hydrothermal vents, *Escarpia* sp. and *Lamellibrachia* sp.2, were discovered in an area of low-temperature diffuse hydrothermal vent flow at the Von Damm site of the Mid-Cayman Rise (MCR), an ultraslow spreading ridge located in the Caribbean Sea [18–21]. Von Damm is an ultramafic system located at a depth of about 2350 m, with end-member venting fluids up to 226 °C that are rich in sulfide, hydrogen, and methane (up to 3.2, 19 and 2.8 mM, respectively [22, 23]. The tubeworms were found at one diffuse vent area of Von Damm, named Shrimp Hole. This site is characterized as a large rock rubble area, with relatively warm fluids (21 to 50 °C), high pH (7.5), and 0.5 mM hydrogen sulfide [20, 21, 24]. Sulfur isotopic data from the Shrimp Hole tubeworms suggested symbioses that utilize hydrogen sulfide from microbial sulfate reduction, similar to other seep tubeworm species [21], while carbon isotopic data indicated use of dissolved inorganic carbon (DIC) source from both seawater and potentially from a thermogenic vent source of hydrocarbon (e.g., methane, ethane, and propane; [21, 22]).

In an analysis of the free-living microbial communities in diffuse vent fluids at Von Damm, MCR vent sites group together, with the exception of Shrimp Hole, which clustered with sediment sites of Guaymas Basin in the Pacific [24]. The most abundant archaeal and bacterial populations at Shrimp Hole were related to *Methanosarcinales* GOM Arc I group, first detected in sediments from a methane seep in the GOM [25], and to sulfate-reducing *Desulfobulbaceae* (Deltaproteobacteria), frequently found in methane-rich sediments together with ANMEs [26], respectively. Other invertebrate taxa sampled at Shrimp Hole, such as *Bathymodiolus* sp. mussels, or *Thyasira* sp. clams, are usually found at seeps [20]. Together, these results indicate the Shrimp Hole site at Von Damm has characteristics of both hydrothermal vents as well as sedimented seeps, thus providing a unique setting for examining microbial endosymbionts in tubeworm hosts.

Here, a cultivation-independent metagenomic approach was used to characterize and compare endosymbionts in the two Mid-Cayman Rise tubeworms species*.* These data, together with electron microscopy and 16S rRNA gene and ITS (internal transcribed spacer) sequence analyses, were used to describe the chemosynthetic endosymbionts and examine how they are related to their Pacific, Gulf of Mexico, and Mediterranean counterparts. These are the first reported metagenome-assembled symbiont genomes for vestimentiferans in the genera *Lamellibrachia* and *Escarpia*, and the first for tubeworms outside the Pacific Ocean.

## Methods

### Sample collection

Tubeworm specimens (Additional file 1) were collected from the Shrimp Hole site on Von Damm vent system on the Mid Cayman Rise (latitude 18° 22.480′ N, longitude 81° 47.841′ W, depth 2375 m) in January 2012 with the Remote Operated Vehicle *Jason 2* (Table 1; [20]). Tubeworms were found in rocky rubble, randomly distributed and co-occurring as single worms rather than in large bushes. Specimens were collected, dissected, and preserved on board ship immediately upon recovery by [20, 18]. Tubeworm species were identified via COI and 16S rRNA gene sequence analyses as *Lamellibrachia* sp. 2 (100% match with symbiont sequences from the Gulf of Mexico) and *Escarpia* sp. (100% match with sequences from the *Escarpia laminata*/*Escarpia southwardae*/*Escarpia spicata* group [20]).

**Table 1:** Summary of the vestimentiferan specimens processed in this study for microscopy, phylogeny, and metagenomics

Species	Specimen ID	Cruise Sample Code	16S rRNA gene haplotype	ITS haplotype	MCR MAG
*Escarpia* sp.	195	J2-616-25	3	60 bp	
	197^a^	J2-616-25	2	60 bp	2
	199^a^	J2-616-25	1		1
	200	J2-616-25	NA		
	689	J2-621-12	1		
*Lamellibrachia* sp2.	192	J2-616-25	NA		
	193	J2-616-25	NA	NA’	
	387^a^	J2-617-38	1		1
	389^a^	J2-617-38	1		1
	391	J2-617-38	4		

*NA* not analyzed due to short sequence (< 700 bp), *NA’* not analyzed due to lack of amplification

^a^Specimen used for metagenomic analysis

For this study, trophosome tissue was dissected from five specimens of each tubeworm species and preserved in 10% formalin for microscopy and in RNALater (Ambion, Inc) for molecular analyses. Tube’s length of *Escarpia* specimens ranged from 270 to 456 mm while the ones of *Lamellibrachia* had length ranging from 180 to 705 mm (Additional file 2).

### Microscopy

Two *Escarpia* (specimen numbers 197,199) and two *Lamellibrachia* (387, 389) tubeworms were examined for the presence of bacterial symbionts (Table 1). For transmission electron microscopy, trophosome tissues were fixed in 10% formalin on board ship, transferred to 70% ethanol after 24 h, and stored at − 80 °C until further processing. Samples were dehydrated in ethanol and a propylene oxide series, and embedded in Spurrs plastic solution (EMS RT 14300, low viscosity). Ultrathin sections were stained with lead citrate and uranyl acetate and examined using a JEM -200CX JEOL transmission electron microscope at the Marine Biological Laboratory (Woods Hole, MA).

### 16S rRNA gene and ITS clone libraries

Molecular characterization of the symbionts was carried out for five specimens of each tubeworm species (including those used for microscopy; Table 1). Trophosome tissue was dissected and placed in RNALater at 4 °C for 24 h, then frozen at − 80 °C until further processing. Total genomic DNA was extracted from 10 mg of trophosome tissue using a MoBio UltraClean Soil DNA Isolation Kit. The 16S rRNA gene and ITS 1 region of the ribosomal operon used to assess the diversity of symbionts within and between individuals from each species were amplified using the bacterial primers 8F (5′–AGA GTT TGA TCC TGG CTC AG–3′) and ITSReub (5′- TGCCAAGGCATCCACC-3′) with an expected amplicon size of approximately 2 kbp [27]. The PCR reaction mixture consisted of 10 μl × 1 Buffer (Promega), 1 μl dNTP (10 mM), 5 μl of forward and reverse primers (10 μM), 0.2 ul of GoTaq DNA polymerase (Promega), 1 μl DNA template, and DEPC H_2_O to 50 μl. Thermocycling conditions consisted of an initial denaturation step at 98 °C for 2 min, followed by 30 cycles of 98 °C for 10 s, 55 °C for 30 s, and 72 °C for 3 min, followed by a final extension at 72 °C for 7 min. PCR products were verified to be the correct size via gel electrophoresis, purified using the Qiagen MinElute PCR Purification Kit following the manufacturer’s instructions and cloned using the TOPO-TA system (Invitrogen). Representative clones (*N* = 24) from each host individual were chosen for full sequencing on an Applied Biosystems 3730XL sequencer using two sets of primers; internally using ITSF (5′-GTCGTAACAAGGTAGCCGTA-3′; [27]) and 1492R (5′- GGTTACCTTGTTACGACTT-3′; [28]) and externally using T3 (5′-ATTAACCCTCACTAAAGGGA-3′) and T7 (5′- TAATACGACTCACTATAGGG-3′).

Full-length clone sequences were processed with an in-house Unix script (available from the authors) that incorporates PHRED, cross_match and PHRAP [29, 30] to translate chromatograms into base calls and associated quality scores, remove vector sequences, and assemble forward and reverse reads into full-length sequences for each of the cloned PCR amplicons. Multiple alignments of the high quality 16S rRNA gene and ITS sequences were performed separately using MUSCLE [31]. Although 24 clones were analyzed per specimen, the number of sequences used for downstream phylogenetic analyses varied, depending on the quality and length of the sequences. Evolutionary analyses were conducted in MEGA [32] with 100 bootstrap replicates. Gaps were removed using the “Complete Deletion” option in MEGA for 16S rRNA gene analysis while all sites were kept for the more variable ITS. Phylogenetic reconstructions were done using Maximum Likelihood (ML) and best model of evolution (i.e., showing the lowest Bayesian Information Criterion in MEGA). 16S rRNA gene and ITS sequences are deposited in GenBank under Accession numbers KY794216- KY794219 and KY795968- KY795976, respectively.

### Metagenomic sequencing and analysis

Trophosome tissue from the same four tubeworm specimens examined with TEM (*Escarpia* MCR 197, 199 and *Lamellibrachia* MCR 387, 389) was analyzed for shotgun sequencing of total community DNA. DNA was sheared to 175 bp using a Covaris S-series sonicator, and metagenomic library construction was completed using the Ovation Ultralow Library DR multiplex system (Nugen) following the manufacturer’s instructions. Metagenomic sequencing was performed on an Illumina HiSeq 1000 and a MiSeq at the W.M. Keck sequencing facility at the MBL. All libraries were paired-end, with a 30 bp overlap, resulting in an average merged read length of 170 bp. Sequence quality trimming and filtering relied upon perfect identity of paired-end read overlaps using the Illumina-utils libraries v.1.4.4 [33]. The four metagenomic datasets were co-assembled using CLC Genomics Workbench (version 7.0.4) (http://www.clcbio.com), discarding contigs smaller than 2000 bp. Each metagenomic dataset was then mapped to the assembled contigs using CLC and a mapping requirement of 95% identity over 80% of the read length and results were exported as BAM files. Subsequent binning analyses were done in a supervised fashion, using both tetranucleotide frequency and coverage for clustering with Anvi’o v1.2.2 (http://github.com/meren/anvio, [32]). Physical dissection and a deep sequencing strategy were used to deal with the high amount of eukaryotic reads, and the eukaryotic vs. bacterial DNA was separated in silico through binning. We generated the Anvi’o holistic display following Anvio’s user manual for Metagenomic Workflow online (http://merenlab.org). The RAST platform was used to infer taxonomy and functions of contigs of bins identified as bacterial as well as to create GenBank files that contain the location and amino acid sequence of genes identified in each genome. We used the pipeline phylosift_v1.0.1 [34] using “phylosift all” with the –isolate and –besthit flags to confirm the taxonomic assignation of the identified draft genomes. RNAmmer 1.2 Server (http://www.cbs.dtu.dk/services/RNAmmer/) was used to retrieve 16S rRNA sequences from single metagenomic assemblies. Average Nucleotide Identity (ANI) was calculated online using the ANI calculator (http://enve-omics.ce.gatech.edu/ani). The metagenome raw sequencing reads are available in the European Nucleotide Archive under Study Accession Number PRJEB19217.

### Free-living microbial community metagenome analysis

To investigate the presence of symbionts in the free-living microbial communities, ten diffuse vent fluids metagenomes from both Piccard and Von Damm sites on the MCR (described in [35]) were mapped to the assembled contigs from the identified symbiont metagenome-assembled genomes (MAGs) using CLC and a mapping requirement of 95% similarity over 80% of the read length.

### Protein clusters generation and pangenomics

Integrated Toolkit for the Exploration of Microbial Pangenomes (ITEP, [36]) was used to profile Genbank files that had been generated using the RAST pipeline [37] from the MAGs identified in this study and five published symbiont genomes assembled from three other tubeworm species. These included two *Ridgeia piscesae* [6], one *Tevnia jerichonana*, and two *Riftia pachyptila* [5] specimens. Briefly, ITEP uses the BLASTP program all vs. all to create a graph of similarities between pairs of proteins and to clusters graphs using the Markov Cluster (MCL) algorithm. Anvi’o v1.2.2 was then used to visualize protein clusters. The ITEP tab-delimited outputs “flatclusters/all_I_2.0_c_0.4_m_maxbit” were transformed into an Anvi’o compatible format using the script “anvi-script-itep-to-data-txt” (https://gist.github.com/meren/4d969b37df61d35bf0baad6baf8092ab). A tree of protein clusters was created based on their distribution across the seven draft genomes using the script “anvi-matrix-to-newick”. The flag –transpose was used to create a sample-order.txt file. Finally, we visualized protein clusters and their distribution across the seven draft genomes in an interactive interface using the script “anvi-interactive”.

### Phylogenetic tree of MAGs

To generate phylogenetic trees of the MAGs, the Phylosift outputs “concatenated protein alignments” located in “alignDir” from each bin were collected and a maximum likelihood phylogenetic trees based on 37 single-copy genes [34] was created with RAxML v.7.2.8 using the “rapid bootstrap” method with 100 bootstraps and the PROTGAMMAWAG protein model of evolution [38]. The outgroup *Sulfurovum* sp. NBC37 was selected to root the tree. Genome information is provided in Additional file 3. We used the “bipartitions” Newick output from RAxML and visualized the trees using the Dendroscope Program [39].

### Single-nucleotide variants in MAGs

The metagenome-assembled genomes represent a microbial population of closely related bacteria in each specimen’s trophosome. In order to retrieve natural variability within that microbial population, single-nucleotide variant (SNV) analysis was performed on each MAG by mapping metagenomic reads to each MAG. SNV analyses were performed using CLC Genomics Workbench 7.0.4 (https://www.qiagenbioinformatics.com/products/clc-genomics-workbench/). Sequences that mapped to each MAG were extracted and re-mapped to that MAG using CLC with a cutoff of 80% identity over 50% of the read. This stringent mapping aimed to aid the identification of reads from the same population and to limit the number of reads from more distantly related members of the microbial community that are part of different populations. CLC’s “Probabilistic Variant Detection” option was used to call variants using (i) a minimum coverage of five reads (i.e., the minimum number of reads aligned to the site to be considered a potential variant), (ii) a variant probability of 80% (of being different from the reference), (iii) a required variant count two (the minimum number of reads supporting the variant), and (iv) “1” as the maximum number of expected alleles (ploidy). The Open Reading Frames (ORFs) used for calling SNVs in each bin were determined by the RAST pipeline [37]. Allele frequencies based on the frequency of the majority allele are reported. In order to minimize the effect of sequencing error, we required all positions to have a minimum coverage of × 10 to be included in SNV analyses, and only counted positions in which the minority allele was represented at least five times.

## Results

### Microscopy and 16S rRNA gene and ITS analyses

As in other vent and seep vestimentiferans, numerous coccoid endosymbionts were observed using TEM in the trophosome tissue of the *Escarpia* and *Lamellibrachia* specimens. Although formalin fixation is not optimal for electron microscopy, the tissues were preserved well enough to see that the trophosome lobules contained numerous coccoid-shaped cells, ranging in diameter from 0.5 to 1.0 μm with cell envelopes resembling those of Gram-negative bacteria. An additional membrane was typically observed surrounding the symbionts, suggesting that as in other vestimentiferans symbioses, the bacteria are contained within membrane-bound vacuoles (Additional file 4A and B).

**Fig. 1:**
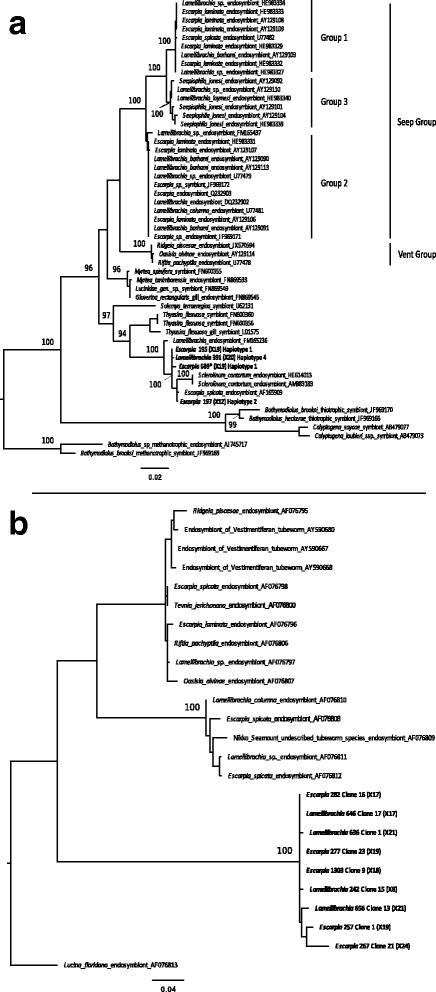
Maximum Likelihood tree showing phylogenetic relationships of MCR and hydrothermal vent vestimentiferan symbionts based (**a**) 16S rRNA and (**b**) ITS sequences using the K2+G+I and K2+G models, respectively. MCR *Escarpia* and *Lamelliabrachia* sp.2 symbiont sequences are highlighted in bold; numbers following names refer to specimen number while numbers in parenthesis, following clone representative sequence, indicate number of clones analyzed per specimen. An asterisk indicates that although *Escarpia* 689 was chosen as the representative sequence for haplotype 1, the latter was shared by *Escarpia* 199 (× 22), 689 (× 19); *Lamellibrachia* 387 (× 20), 389 (× 21) individuals. For 16S, ca. 1360 bp nucleotides were analyzed with sequences < 700 bp were excluded. The tree was rooted with methanotrophic *Bathymodiolus* mussel symbiont as an outgroup. ITS analysis included ca.575 nucleotides. The tree was rooted with the symbiont from *Lucina floridana*, a marine bivalve (clam) as an outgroup. Accession numbers follow host names and numbers at nodes (listed above 85) indicate the proportion of occurrences in 100 bootstrap replicates. Seep groups 1, 2 and, 3 and Vent group correspond to vestimentiferans symbionts highlighted in Thiel et al. [14]

The phylogenetic relationships of the tubeworm symbionts were characterized by amplification, cloning, and sequencing of the 16S rRNA gene and ITS from the trophosome tissue of ten tubeworm specimens (five of each species; Table 1). Three specimens (*Escarpia* MCR 200; *Lamellibrachia* MCR 192, 193) were removed from the 16S rRNA gene analysis due to short (< 700 bp) sequences. The 16S rRNA gene sequences of each of the seven remaining individuals had > 99% sequence identity over the entire length (ca.1370 bp), thus likely representing one 16S rRNA gene, or closely related haplotypes per individual. An analysis of these sequences showed four different 16S rRNA gene haplotypes that diverge by a maximum of 0.7% (Fig. 1a). Haplotype 1 was shared by *Escarpia* (199, 689) and *Lamellibrachia* (387, 389) individuals, while *Escarpia* 197, 195, and *Lamellibrachia* 391 were represented by haplotypes 2, 3, and 4, respectively (Fig. 1a).

Phylogenetic analyses of the 16S rRNA gene revealed the endosymbiont of *Escarpia spicata* from the Guaymas Basin is the closest relative (AF165909; 99% sequence identity), with only 3 nucleotides’ differences over a total alignment length of 1368 bp to haplotype 2. Closely related sequences to haplotypes 1, 3, and 4 are from the same endosymbiont of *Escarpia spicata* and another vestimentiferan (*Sclerolinum contortum*) endosymbionts (HE614013 from the Gulf of Mexico; 99% sequence identity, with a maximum of 18 nucleotides differences over a total alignment length of 1367 bp, and AM883183 from Norway, 99% sequence identity, maximum18 nucleotides differences over a total alignment length of 1367 bp) (Fig. 1a). A sister group consisted of symbionts from the marine bivalve *Thyasira flexuosa* (FN600359.1; 96% identity, 53 nucleotide differences with haplotype 1 over a length of 1359 bp). The endosymbionts of hydrothermal vent tubeworms *Riftia pachyptila*, *Oasisia alvinae,* and *Ridgeia piscesae* (“Vent Group”) and the majority of the seep tubeworm symbionts (groups 1, 2, 3) were more distantly related (Fig. 1a; groups as described by Thiel et al. [14]).

Similar to the 16S rRNA gene analysis, the symbiont ITS sequences of each individual worm (nine specimens) had at least 99% sequence identity over 577 bp; thus, each individual was represented by a single or closely related phylotype. One specimen (*Lamellibrachia* MCR 193) was removed from the ITS analysis due to lack of amplification. When compared to each other, the different ITS phylotypes showed more than 98% sequence identity, with the exception of individuals *Escarpia* 195 and 197 which had a 60 bp insert between the sequences encoding the two tRNAs, Ile and Ala. Based on the ITS phylogenetic analysis, the endosymbionts formed a separate well-supported clade that diverged from other seep or vent vestimentiferan tubeworm endosymbiont sequences (Fig. 1b).

### Metagenomic analysis

*Escarpia* 197 and 199 and *Lamellibrachia* 387 and 389 were selected as representative specimens following 16S rRNA gene and ITS analyses for metagenomic sequencing (Table 2). Shotgun sequencing of total community DNA yielded a total of more than 22 and 11 million of high-quality sequences for *Escarpia* 197 and 199, respectively, and more than 20 and 24 million of high-quality sequences for *Lamellibrachia* 387 and 389, respectively (Table 2).

**Table 2:** Number of high-quality filtered reads for metagenomes (after Illumina-utils merge and filter) and mapping statistics

Species	Specimen ID	High-quality sequences	Read % recruited to co-assembly
*Escarpia* sp.	197	22,810,633	19.2
*Escarpia* sp.	199	11,197,396	29.0
*Lamellibrachia* sp2.	387	20,585,832	32.0
*Lamellibrachia* sp2.	389	24,013,669	15.4

**Fig. 2:**
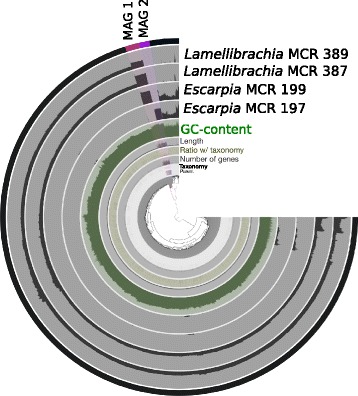
Static image from the Anvi’o interactive display for the *Escarpia* and *Lamellibrachia* tubeworm datasets with two symbiont genome bins highlighted in red (MCR MAG 1) and purple (MCR MAG 2). The inner clustering dendrogram displays the hierarchical clustering of contigs based on their sequence composition, and their distribution across samples (i.e., differential coverage). Anvi’o divides a contig into multiple splits if it is longer than 20,000 bps and each tip on this hierarchical clustering represents a split. Auxiliary layers from inside to outside report information about contigs stored in the contig database (parent marks splits that originate from the same contigs with gray bars, RAST taxonomy that shows the consensus taxonomy for each open reading frame (ORF), number of genes shows the number of open reading frames, ratio with taxonomy shows the proportion of the number of ORF with a taxonomical hit in a given split, length shows the actual length of a given split, and GC-content). The four next view layers report information about contigs across samples stored in the profile database. The most outer layer shows the genome bins. The eukaryotic component is highlighted in black

The co-assembly of the four metagenomes yielded 30,603 contigs > 2 kb (average contig length 3131 bp; max contig length 233,535 bp) for a total assembly size of 95,810,717 bp. Anvi’o was used to identify draft genomes of bacterial origin [40]. Clustering of contigs with respect to their sequence composition and coverage patterns across the four symbiont metagenomes revealed two distinct bins that contained 4,260,814 bp and 4,643,056 bp with completion/redundancy scores of 90.3/4.8% and 97.9/4.8%, hereafter referred to MCR MAG 1 (200 contigs) and MCR MAG 2 (82 contigs), respectively (Fig. 2). This calculation is based on the single occurrence of 139 single-copy genes (SCG) identified from the collection of Campbell et al., [41] implemented in Anvi’o [40]. More than 90% completion and less than 10% redundancy based on this collection of bacterial single-copy core genes suggest high completion of the bins (http://merenlab.org/2016/06/09/assessing-completion-and-contamination-of-MAGs/). Minimum information about both MCR MAGs is provided in Additional file 5 following Bowers and colleagues [42]. MCR MAG 1 was identified in *Escarpia* 199 and *Lamellibrachia* spp. 387 and 389 (with mean coverage of ~ 245X, 206X and 18X, respectively), while MCR MAG 2 was identified in *Escarpia* 197 (with mean coverage of ~ 177X). The two MAGs showed an Average Nucleotide Identity (ANI) of 86.4% and represented 13.6% of the total assembly, with the remaining 86.4% representing eukaryotic host (highlighted in black in Fig. 2). The two MAGs did not recruit any reads from the ten diffuse vent fluids metagenomes from both Piccard and Von Damm vents at the MCR (described in [35]).

**Fig. 3:**
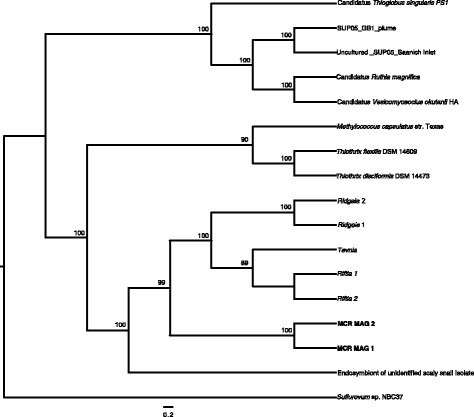
Maximum Likelihood tree showing phylogenetic relationships of MCR (MAG 1 and MAG 2), other vent vestimentiferans symbionts, and *Gammaproteobacteria* isolates. Phylogenies are based on 37 single copy gene sequences from symbiont metagenomes and isolate genomes. Phylosift [34] was used to identify, concatenate, and align universal marker genes. Phylogenetic analysis included 6449 nucleotides. MCR MAG 1 was detected in both MCR tubeworms, *Lamellibrachia* sp. 2 (387 and 389) and *Escarpia* sp. (199), while MCR MAG 2 was only found in *Escarpia* sp. (197). ML bootstrap (above 85) are indicated at nodes and NCBI Tax ID, Genbank Accession number and references are provided in Additional file 3)

The MAGs were compared to the best matching reference genomes using the best-hit function implemented in RAST and assigned to *Gammaproteobacteria*. A phylogenetic tree based on 37 universal single-copy genes from *Gammaproteobacteria* [34] showed that the Mid-Cayman Rise tubeworm endosymbionts formed a well-supported clade apart from the symbionts of the other species of tubeworms (Fig. 3). Endosymbionts of the three vent tubeworm species genera clustered together, with separate monophyletic clades for *Ridgeia*, *Riftia*, and *Tevnia*, respectively, in agreement with previous studies. ANI values between MCR MAG 1 or 2 and either *Riftia*, *Tevnia* or *Ridgeaia* endosymbiont’s genomes were less than 75%. Although 16S rRNA gene sequences were not retrieved from the co-assembly, one 16S rRNA gene sequence was extracted from each MAG individually. These sequences were identical to the haplotype sequences retrieved from the clone library sequences; haplotype1 was shared between *Escarpia* 199, *Lamellibrachia* 387, and *Lamellibrachia* 389 and haplotype 2 was found in *Escarpia* 197. In addition, SNV density/kb and average allele frequency were measured in each MAG from each specimen. We observed very few SNVs in each of these *Gammaproteobacteria* bins (less than 0.5 SNPs/kbp) and low to middle allele frequency (Additional file 6).

**Fig. 4:**
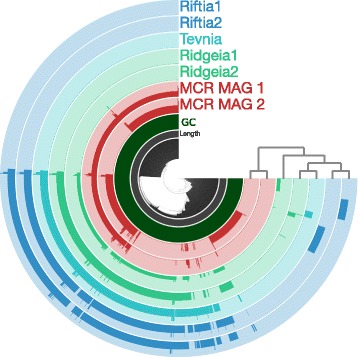
Distribution of protein clusters (PC) in the seven metagenome-assembled genomes (MAGs) from *Ridgeia piscesae* [6], *Tevnia jerichonana*, *Riftia pachyptila* [5] and from the MCR specimen studied herein. In this figure, circles represent MAGs. Radius represent PC. A bar in the genome layer represents the occurrence of a PC. The 7679 PC are clustered based on their distribution among the seven tubeworm genomes (inner dendogram). The organization of the seven genomes is defined by the shared PC (dendogram on the right)

Because bacterial marker single-copy genes (i.e., core genes) do not necessarily mirror variation in the rest of the genome, a pangenomic analysis was also conducted and protein clustering was used to characterize endosymbiotic tubeworm MAGs based on their entire gene content. Downstream automated open-reading frame identification and functional assignment predicted 3910 protein coding sequences (CDS) for MCR MAG 2, whereas 4292 CDS were determined for MCR MAG 1. The pangenomic analysis included the two identified MAGs from this study, the five assembled symbiont genomes of the vent vestimentiferan *Ridgeia piscesae* (2 genomes, [6]) as well as *Tevnia jerichonana,* and *Riftia pachyptila* (2 genomes, [5]). ITEP identified 7679 protein clusters of Unique Protein Encoding Genes (PEG) across all genomes. One thousand three hundred thirty-four protein clusters (PC) were shared across all genomes, while 1263 PC were specific to the MCR *Lamellibrachia* and *Escarpia* species (Fig. 4) and 1180 and 949 were unique to MCR MAG 1 and MCR MAG 2, respectively. The distribution pattern of genomes based on protein clusters shows the two MCR endosymbiont MAGs cluster apart from the other metagenome-assembled genomes (*Riftia*, *Tevnia* and *Ridgeia*).

Key genes involved in major metabolic pathways, as well as genes involved in symbiont–host interactions and protein secretion, were identified in the two MCR endosymbiont MAGs and compared to the *Riftia*, *Tevnia*, and *Ridgeia* symbiont metagenome-assembled genomes (Additional file 7). As with the MCR tubeworms, none of the tubeworm symbiont genomes used in these comparative analyses are closed; thus, a definitive list of all symbiont genes could not be made, and we acknowledge this limitation. However, the high depth of sequence coverage and the analysis based on 37 core bacterial gene markers for all seven symbiont genomes suggest that most gene-coding regions were detected. Genes that were only identified in both MCR MAGs and not in any of the other genomes are shown in Table 3. Genes for both the Calvin Benson Bassham (CBB) cycle and the reductive tricarboxylic acid (rTCA) cycle were found in the two MCR MAGs, as well as in all other endosymbiont genomes. The gene encoding phosphoribulokinase (*cbb*) was shared across all genomes, and the gene for ribulose bisphosphate carboxylase (form II RubisCO, *cbbM*), the CO_2_ fixing enzyme of the CBB cycle, was detected in at least one individual of each species. Genes coding for 2-oxoglutarate oxidoreductase, a key gene in the rTCA cycle, were also detected. In addition, several genes for C_4_-dicarboxylate transport systems, for the uptake of C_4_ molecules such as malate and succinate, were identified across all genomes. Genes for potential heterotrophic metabolism were also identified in all seven genomes, including those coding for the phosphoenolpyruvate (PEP)-dependent phosphotransferase system (*pts*), glycolysis (e.g., glucose-6-phosphate isomerase), the Krebs cycle (e.g., citrate synthase), and several abc transporters for the uptake of peptides and lipopolysaccharide. Further, several additional abc transporters were detected only in the MCR genome bins (including those for branched-chain amino acids, peptides, oligopeptide/dipeptides, and nucleosides).

**Table 3:** Genes identified in both MCR MAGs and not in the other genomes

Metabolism	Annotation
Formate Oxidation	NAD-dependent formate dehydrogenase alpha subunit
Oxidation of hydrogen	NAD-reducing hydrogenase subunit hoxf (ec 1.12.1.2) NAD-reducing hydrogenase subunit hoxh (ec 1.12.1.2) NAD-reducing hydrogenase subunit hoxu (ec 1.12.1.2) NAD-reducing hydrogenase subunit hoxy (ec 1.12.1.2)
ABC transporter	Phosphate abc transporter, periplasmic phosphate-binding protein psts (tc 3.a.1.7.1) Branched-chain amino acid abc transporter, amino acid-binding protein (tc 3.a.1.4.1) Glycerol-3-phosphate abc transporter, permease protein ugpa (tc 3.a.1.1.3) abc transporter atp-binding protein uup Branched-chain amino acid abc transporter, atp-binding protein abc transporter, atp-binding protein Oligopeptide/dipeptide abc transporter, atp-binding protein abc transporter, atp-binding protein Nucleoside abc transporter, atp-binding protein Uncharacterized abc transporter, auxiliary component yrbc Glycerol-3-phosphate abc transporter, permease protein ugpe (tc 3.a.1.1.3) Branched-chain amino acid abc transporter, amino acid-binding protein (tc 3.a.1.4.1) Branched-chain amino acid abc transporter, periplasmic substrate- binding protein Phosphate abc transporter, periplasmic phosphate-binding protein psts (tc 3.a.1.7.1) Possible abc transporter subunit abc transporter, permease protein Branched-chain amino acid abc transporter, amino acid-binding protein (tc 3.a.1.4.1) Putative abc transporter protein abc transporter atp-binding protein abc transporter permease protein Peptide abc transporter, permease component Dipeptide-binding abc transporter, periplasmic substrate-binding component (tc 3.a.1.5.2) abc transporter, permease protein, putative Nucleoside abc transporter, permease protein 2 Nucleoside abc transporter, permease protein 1 abc transporter, periplasmic substrate-binding protein Spermidine putrescine abc transporter permease component potc (tc_3.a.1.11.1) abc transporter, periplasmic spermidine putrescine-binding protein potd (tc 3.a.1.11.1)
Oxidative stress response	Catalase (ec 1.11.1.6)
Host infection, environmental defense, and secretion system	abc-type nitrate/sulfonate/bicarbonate transport systems, periplasmic components abc-type phosphate/phosphonate transport system periplasmic component abc-type polar amino acid transport system, atpase component abc-type transport system involved in multi-copper enzyme maturation, permease component abc-type tungstate transport system, atp-binding protein abc-type tungstate transport system, periplasmic binding protein abc-type tungstate transport system, permease protein Exopolysaccharide production protein pss General secretion pathway protein a General secretion pathway protein d General secretion pathway protein h General secretion pathway protein i General secretion pathway protein j General secretion pathway protein k General secretion pathway protein l General secretion pathway protein m General secretion pathway protein n Membrane fusion protein of rnd. family multidrug efflux pump rnd. multidrug efflux transporter; acriflavin resistance protein Secretion protein hlyd precursor Type i secretion system, outer membrane component lape # agga Type i secretion system, membrane fusion protein lapc Type i secretion system atpase, lssb family lapb Type i secretion outer membrane protein, tolc precursor Type i secretion system atpase, lssb family lapb Type i secretion system, membrane fusion protein lapc Type i secretion system, outer membrane component lape # agga Type ii and iii secretion system family protein

MCR MAGs’s unique gene subunits are not listed herein when other subunits belonging to the same gene were detected in other genomes

The genetic potential for sulfur oxidation of the different endosymbionts was identified in all MAGs by the presence of genes coding for dissimilatory sulfite reductase (*dsrAB*), an enzyme of both sulfur assimilatory and dissimilatory sulfate reduction (APS), as well as sulfate thiohydrolase (*soxBXYZ*), an essential component of the Sox multienzyme complex [43]. Genes for the complete respiration of nitrate to dinitrogen gas were also detected in all MAGs, including nitrate reductase (*nar*), nitrite reductase (*nir*), nitric oxide reductase (*nor*), and nitrous oxide reductase (*nos*). In addition, the potential for nitrogen assimilation was confirmed in all MAGs, with the presence of genes coding for glutamate synthase [44]. The genes encoding an uptake hydrogenase involved in the oxidation of molecular hydrogen ([NiFe] hydrogenase *hyaA*, *hyaB*, *hybC*) for energy generation was found in all MAGs. In addition, we detected an additional hydrogen oxidation pathway, as shown by the presence of the full set of genes coding for the bidirectional hydrogenases “NAD^+^-reducing hydrogenase subunits *hoxHYUF*” in the MCR MAGs only. A BLAST analysis showed these Hox genes were closely related to those from the free-living *Gammaproteobacteria Sedimenticola selenatireducens* [45]. Finally, a gene fragment encoding an NAD+-dependent formate dehydrogenase was also found exclusively in the two MCR symbiont MAGs.

In addition to metabolic pathways, we identified oxidative stress response genes in all endosymbiont MAGs, including genes encoding superoxide dismutase, alkyl hydroperoxide reductase subunit c-like protein, and thioredoxin reductase. A gene encoding a catalase was detected for the first time in tubeworm symbiont genomes in both MCR MAGs. Several genes encoding multidrug efflux pumps involved in the export of antibiotics and other toxic compounds from the cell were also detected across all seven genomes. We found several ABC transporters annotated as toxin or multidrug exporters that likely represent symbiont defense mechanisms against the host. Although type VI secretion system *vgrg* protein has only been in *Ridgeia*, several genes encoding secretion systems such as type I, II secretion systems, and general secretion pathway proteins *a-n* were found across all genomes. In addition, abc-type antimicrobial peptide transport system were found in all genomes. The autolysis response regulater *lytr* was found in all genomes. A gene for the exopolysaccharide production protein (*pss)* was only found in the MCR MAGs.

## Discussion

This study allowed the first tubeworm endosymbiont metagenome-assembled genomes from either *Lamellibrachia* or *Escarpia* species to be characterized and compared to other tubeworm endosymbiont genomes. Earlier studies have shown genetic differences between tubeworm endosymbionts between seep and vent species at the 16S rRNA gene level [9, 14]. Our phylogenetic and genomic data show that *Lamellibrachia* and *Escarpia* symbionts from the Mid Cayman Rise share highly similar endosymbionts, which differ from other tubeworm endosymbionts, and in particular, from the well-described vent tubeworm endosymbionts from the Pacific (Figs. 1, 3 and 4). When compared to other seep-like symbionts, the tubeworm endosymbionts from the MCR formed a well-defined and separate clade at the ITS level and were distantly related from the seep tubeworms groups described by Thiel et al. [14] at the 16S rRNA gene level. They showed high sequence similarity with other symbionts. For instance, haplotype 2 showed 99% sequence similarity to endosymbionts from *Escarpia spicata* from the Guaymas Basin and was also closely related to the symbionts of the tubeworm *Sclerolinum contorum*. However, with more than 5% divergence from both *Rifta* and *Ridgeia* endosymbionts at the 16S rRNA gene level, it is possible that the differences in symbionts between and within seep and vent tubeworm have been underestimated in past studies and that different species occur at seep and vent habitats.

The metagenome-assembled genome results are consistent with the 16S rRNA gene and ITS analyses, indicating the MCR MAGs are distinct from all other sequenced tubeworm endosymbionts based on both phylogenetic analysis of concatenated core genes as well as protein clusters (Figs. 1 and 3). Within the MCR tubeworms, we identified MCR MAG 1, shared by both *Lamellibrachia* individuals and one *Escarpia* individual, as well as MCR MAG 2, which was only found in the other *Escarpia* individual. The two MCR MAGs showed an ANI of 86.4%, and MCR MAG2 had both a different 16S rRNA gene haplotype (haplotype 2), as well as an ITS region that contained a 60 bp insertion, as detected via cloning and sequencing, confirming the presence of multiple MCR symbionts. The whole genome analysis showed 1334 protein clusters shared across the seven genomes while 1263 were unique to the MCR MAGs, including whole pathways (Table 3). Despite nucleotide-level differences at the 16S rRNA gene, ITS, and whole genome level, there was little difference in functional capacity between the two genomes. While 1180 and 949 PC were found as unique to MCR MAG 1 and MCR MAG 2, respectively, we did not identify any specific pathway in one or the other. This implies that similar selective constraints act on both endosymbiont populations, in agreement with previous studies that focused on endosymbionts from different host species occurring in the same geographic area. For instance, the gene content and sequences of *Riftia* and *Tevnia’s* endosymbionts from the East Pacific Rise were highly similar to each other, despite the fact that the two species thrive in distinct vent geochemical conditions [5]. The authors suggest that host species are able to attenuate differences in the environmental conditions experienced by each individual, thus providing their endosymbionts with similar microenvironments [5].

Key genes previously described in vent tubeworm metagenome-assembled genomes, including genes for the CBB and the rTCA cycle, as well as sulfur, nitrogen, and hydrogen oxidation, were identified from the MCR MAGs, underlying some core and shared functions between all tubeworm symbionts, independent of their geographic location [5, 8, 14]. These results are in agreement with carbon and sulfur isotopic data from Von Damm, which indicate sulfide-oxidizing chemoautotrophic endosymbionts for the tubeworms [21]. Bennett and colleagues [21] suggested that despite high level of hydrogen sulfide in the venting fluids at Von Damm, the tubeworm endosymbionts rely on sulfide from microbial sulfate reduction, similar to seep tubeworm species.

Given the high concentrations of hydrogen at Von Damm (19 mM), it is thus available to the symbionts as an alternate energy source. We found genomic evidence for hydrogen oxidation using the Hox gene pathway, which has not previously been identified in tubeworm endosymbionts nor in any other hydrothermal vent symbioses, to our knowledge. The full set of Hox genes encoding the bidirectional hydrogenases “NAD-reducing hydrogenase” was identified in all MCR tubeworm endosymbiont MAGs. First described in the hydrogen-oxidizing chemosynthetic *Betaproteobacteria Cupriavidus necator* (*Ralstonia eutropha)*, the [NiFe]-hydrogenase of group 3d (as defined in the phylogenetic classification of Vignais and colleagues [46]), the Hox genes have now been found in several Archaea and Bacteria. A BLAST analysis showed the Hox genes were closely related to those from free-living *Gammaproteobacteria*, suggesting these could have been acquired from close relatives, although we do not have evidence for horizontal gene transfer (HGT)*.* Given the endosymbiont MAGs also possess the commonly found Group 2a [NiFe]-hydrogenase (i.e., uptake hydrogenase, *hupL*) which occurs in other chemosynthetic symbioses including *Riftia* [47], it is not clear how these pathways are being utilized, concomitantly or separately depending on the conditions, requiring further investigation.

Further, based on the genomic evidence, the MCR tubeworm symbionts have the capacity to utilize organic compounds. The tubeworm endosymbionts may oxidize formate to carbon dioxide, catalyzed by NAD+-dependent formate dehydrogenase (*fdhA*), which has not previously been reported in tubeworm endosymbionts. Formate measurements in mixed fluids at Von Damm indicate concentrations up to 669 μmol kg^−1^, which are similar to those measured at the ultramafic hosted Lost City site (up to 158 μmol kg^−1^; [48]). The presence of this gene in the tubeworm endosymbionts may be due to the adaptation of the organisms to a formate-rich system. In addition, NAD+-dependent formate dehydrogenases are also important in methylotrophic microorganisms [49], which can use reduced one-carbon compounds, such as methanol or methane, as both an electron donor and a carbon source, with oxygen as the final electron acceptor. Bennett and colleagues [21] inferred from stable carbon isotopes that some seep-associated fauna at Von Damm rely on thermogenic hydrocarbons from the venting fluids for their carbon source; thus, the potential ability for tubeworm endosymbionts to oxidize organic substrates such as formate, or single carbon compounds indicates they may be able to switch from an autotrophic to a heterotrophic metabolism, possibly while free-living. Such metabolic versatility was already suggested in the *Riftia* tubeworm based on the capacity to transport and oxidize organic compounds [8], and the present study adds new evidence that tubeworms endosymbionts can adapt to very different sources of carbon and energy. The presence of peptide, sugar and lipid transporters, detected across all seep and vent metagenomes, and in particularly, in relatively high abundances in the MCR MAGs, further illustrate this metabolic versatility. In light of these and previous studies, and given the venting fluids at Von Damm are rich in carbon compounds such as formate and methane, we hypothesize that the tubeworm endosymbionts of the Mid-Cayman Rise tubeworms are chemoautoheterotrophic bacteria, rather than purely chemosynthetic bacteria. Gene expression studies would be required to infer if heterotrophy is more important or restricted to free-living symbionts.

We also identified genes within the MCR MAGs that provide insight into host-symbiont interactions. For example, we found a gene that may be involved in extracellular biofilm matrix formation through the biosynthesis of exopolysaccharides (*pss*). In addition, secretion systems detected in all tubeworm endosymbiont genomes such as the hlyd family secretion protein (*hlyD*), or the secretion systems type i, ii are used by bacteria to infect their host cells via a process called cell adhesion [50]. Although some molecular mechanisms for host-symbiont interactions have been described, they share striking similarities with pathogenic mechanisms. In a previous endosymbiont tubeworm metagenomic study, Gardebrecht and colleagues [5] showed the presence of genes involved in infection, biofilm formation, tissue lysis, and virulence related to those found in the pathogens *Burkholderia pseudomallei*, *Staphylococcus aureus*, and *E. coli*. Likewise, Sayavedra and colleagues [51] showed that mussel symbionts express toxin-related genes to interact with their hosts, highlighting mechanisms common to both pathogenic infections and beneficial host–symbiont interactions. In all the tubeworm endosymbiont genomes, we identified several ABC transporters annotated as toxin or multidrug exporters that represent pathogen-type defense mechanisms for the symbiont to protect itself from host defenses after infection, as well as many multidrug efflux pumps that can export toxin compounds present in the hydrothermal environment or from the cell. We also found autolysins genes in all of the endosymbiont genomes that are important in surface adhesion in *Lactococcus lactis* and in the pathogenic properties of *Streptococcus pneumonia* [52]. The presence of antimicrobial peptides (AMP) genes in all genomes suggest the symbionts protect the host or themselves against pathogens, parasites, parasitoids, or predators, as described in other animals [53]. A gene coding for the antioxidant enzyme catalase was found exclusively in the MCR endosymbiont MAGs, suggesting the ability of symbionts to break down hydrogen peroxide (H_2_O_2_) to water and oxygen. This gene has not been identified in tubeworm endosymbiont genomes previously, and its presence in the two MAGs suggests the MCR symbionts have unique enzymatic defenses against oxygen radical damage [54].

Taken together, our data show high levels of metabolic potential and versatility in the genomes of tubeworm endosymbionts at the MCR. The potential capacity of *Lamellibrachia* and *Escarpia* symbionts to use an extensive range of energy sources including sulfide and hydrogen to fuel chemosynthesis and organic matter (e.g., hydrocarbons) for heterotrophic metabolism may enable the host to exploit the wide range of environmental conditions in the ultramafic, sulfide, hydrogen, and methane rich venting fluids at Von Damm. This high metabolic diversity is consistent with the relatively large size of the genome bins reconstructed herein (more than 4 Mbp each). The vestimentiferan endosymbiont genomes obtained from “strict” (i.e., bare-rock in opposition to seep) vent systems habitats have slightly smaller sizes (i.e., *Ridgeia* 1 symbiont 3.44 Mbp; *Ridgeia* 2 symbiont 3.42 Mbp; Tevnia symbiont 3.64 Mp; *Riftia* 1 symbiont 3.48 Mbp; *Riftia* 2 symbiont 3.71 Mbp; “*Candidatus* Endoriftia persephone” 3.20 Mbp; see [6]). The fact that we did not obtain a single contig for each of the metagenome-assembled genomes reconstructed for this study could be due to the presence of repeated sequences across the genomes, which can end up in assembly break. Future studies using long read sequencing strategies such as MinION from Nanopore could help closing these genomes. The observed high metabolic diversity raises the question of whether a single strain or several partners are responsible for the observed diversity. Our results do not eliminate strain-level variation in the symbiont communities used to create the metagenome-assembled genomes. Genomic heterogeneity was shown in vent mussel symbiont populations that either possess or lack a key gene cluster, suggesting specialized rather than generalists symbionts [55]. The low SNV density in each of the MAGs, however, suggests a relatively low number of strains in each symbiotic bin, and therefore a low symbiont heterogeneity. Although we could not estimate the number of strains within each MAG, these genome bins (or genome populations) are relatively homogenous populations. Further, the low allele frequency (suggesting even abundance of the variants) in bins with high coverage (up to 245X) suggests abundant populations that are almost clonal. These data make the presence of metabolic specialists unlikely and suggest that individual symbionts from the MCR are able to accomplish a wide array of functions.

Together, the marked gene content and sequence dissimilarity (at the rRNA gene and whole genome level, with less than 75% ANI values) between hydrothermal and the seep endosymbionts studied herein suggest endosymbionts from the MCR belong to a novel tubeworm endosymbiont genus. We introduce the names *Candidatus* Vondammii proteani (i.e., named after the feature of sea-god Proteus, a figure of “flexibility, versatility and adaptability”) and *Candidatus* Vondammii crypti to distinguish MAG1 and MAG2, respectively.

## Conclusions

The genomic analysis coupled with phylogenetic and microscopic characterization of Mid-Cayman Rise *Escarpia* and *Lamellibrachia* endosymbionts sheds new light on tubeworm symbionts, extending both the potential habitats and metabolic flexibility of deep-sea hydrothermal vent and seep symbioses.

## Additional files

Additional file 1: Tubeworms recovered from Shrimp Hole at Von Damm, Mid-Cayman Rise with Remote Operated Vehicle *Jason 2*. Photographs: A, B In situ images of tubeworms on basalt C, D. Tubes of *Escarpia* and *Lamellibrachia,* showing anterior curved roots and higher magnification of tube opening, respectively, EF, *Escarpia* and GH, *Lamellibrachia* specimens extracted from tubes showing*,* branchial plumes (E-G) and trophosome (F, H). Photographs credit: Woods Hole Oceanographic Institution. (PDF 21694 kb)

Additional file 2: Measurements of *Escarpia* sp. and *Lamellibrachia* sp. 2 MCR vestimentiferan specimens used in this study. (XLSX 11 kb)

Additional file 3: Isolate and vestimentiferan symbiont metagenome information for taxa included in Fig. 3 including organism name, NCBI TaxID, Genbank Accession number and reference. (XLSX 10 kb)

Additional file 4: Transmission electron micrographs of trophosome tissue of Mid-Cayman Rise vestimentiferans, *Escarpia* sp.(A) and *Lamellibrachia* sp.2 (B). showing coccoid endosymbionts with cell envelopes (ce) resembling those of Gram negative bacteria. The symbionts are typically surrounded by an additional membrane (arrows) suggesting they are contained within membrane-bound vacuoles (v) in host cells as in other vestimentiferans symbioses (PDF 1793 kb)

Additional file 5: General genome metadata and quality information for MCR MAGs 1 and 2 assembled from *Escarptia* sp. and *Lamellibrachia* sp. 2. (XLSX 39 kb)

Additional file 6: Relationships between Single Nucleotide Variant (SNV) density/kb, SNV average allele frequency, and mean coverage for each MCR genomic bin in the four different tubeworm individuals (L and E indicates *Lamellibrachia* and *Escarpia*, respectively, with specimen number following). Size of bubble indicates coverage, with the largest bubble corresponding to a coverage of 245X (E-199), the smallest one to 18X (L- 389) and the intermediate ones to 177X (E-197) and 206X (L-387). (PNG 46 kb)

Additional file 7: Genes involved in major metabolic pathways, symbiont–host interactions, and protein secretion in MCR MAGs 1 and 2 in comparison with the *Riftia*, *Tevnia,* and *Ridgeia* symbiont metagenomes. Gene clusters were identified by ITEP and annotated with RAST. The last column indicates gene clusters only present in the MCR MAG bins (value =1). (XLSX 39 kb)

## Abbreviations

ANI: Average Nucleotide Identity
ITS: Internal transcribed spacer
MAG: Metagenome-assembled genome
MCR: Mid-Cayman Rise
