# Supplementary figures and images for: Metagenomic investigation of vestimentiferan tubeworm endosymbionts from Mid-Cayman Rise reveals new insights into metabolism and diversity
Source: Microbiome. 2018 Jan 27;6:19. doi: 10.1186/s40168-018-0411-x (PMC5787263; doi:10.1186/s40168-018-0411-x)

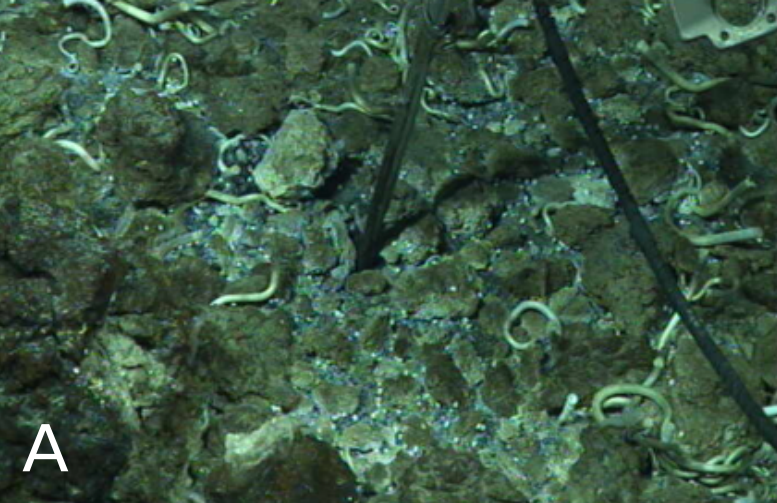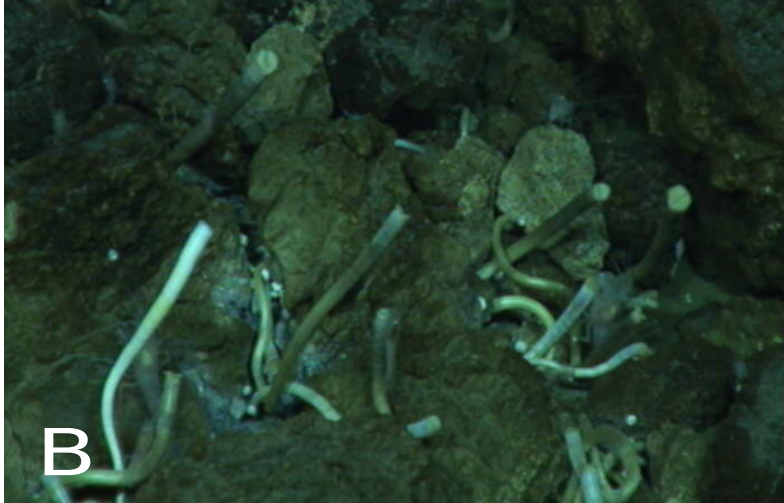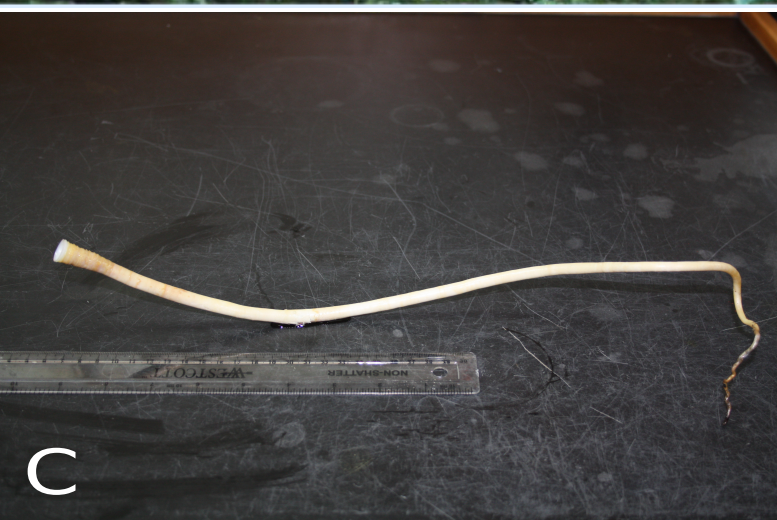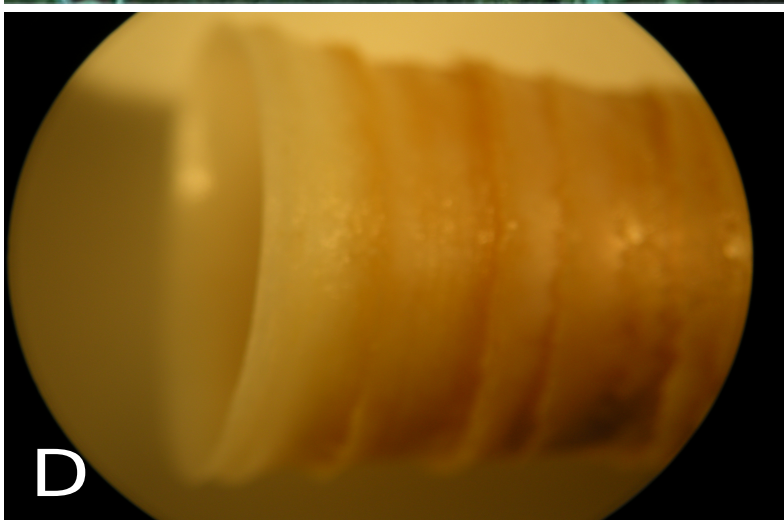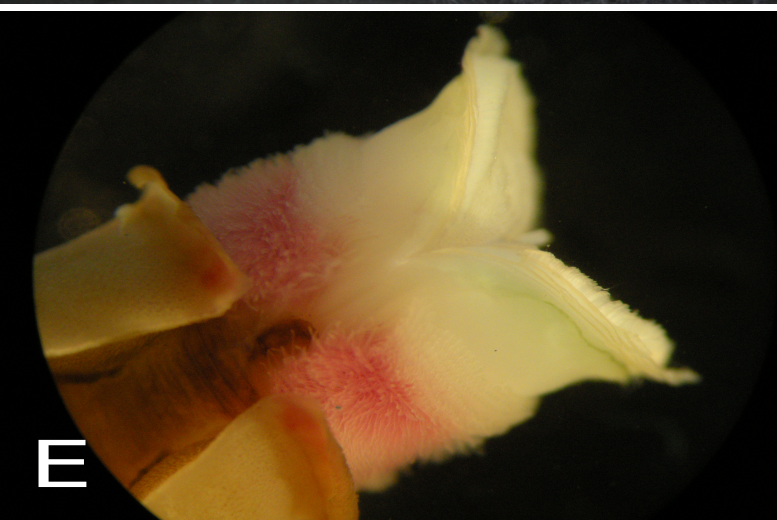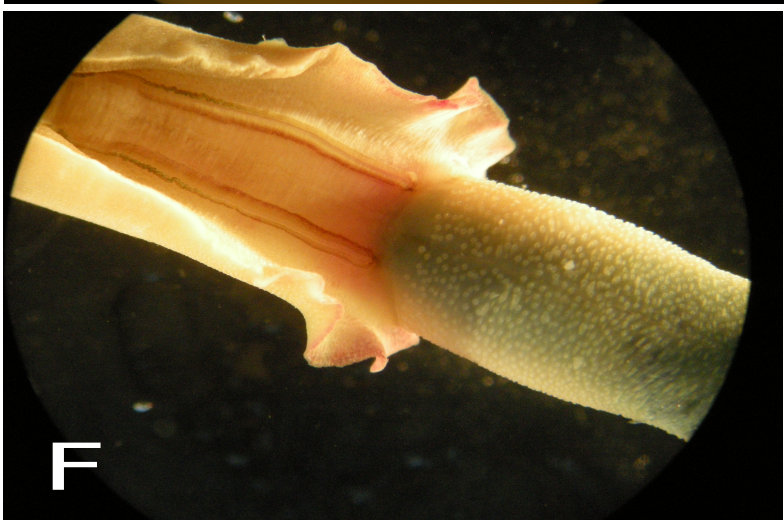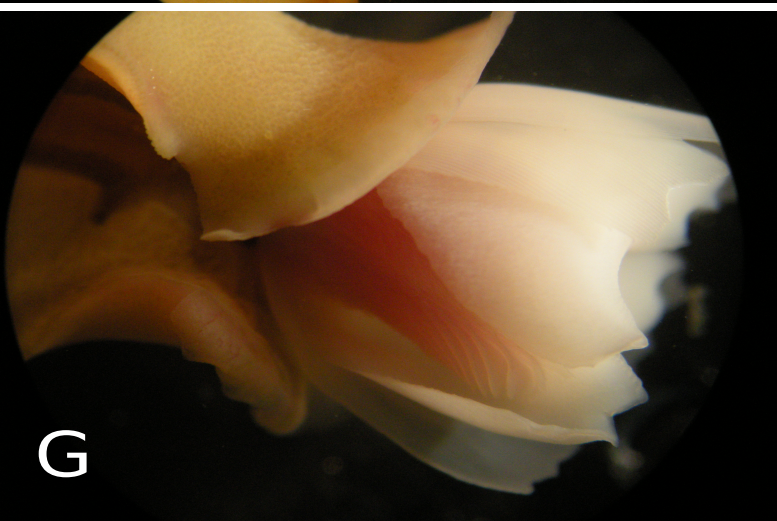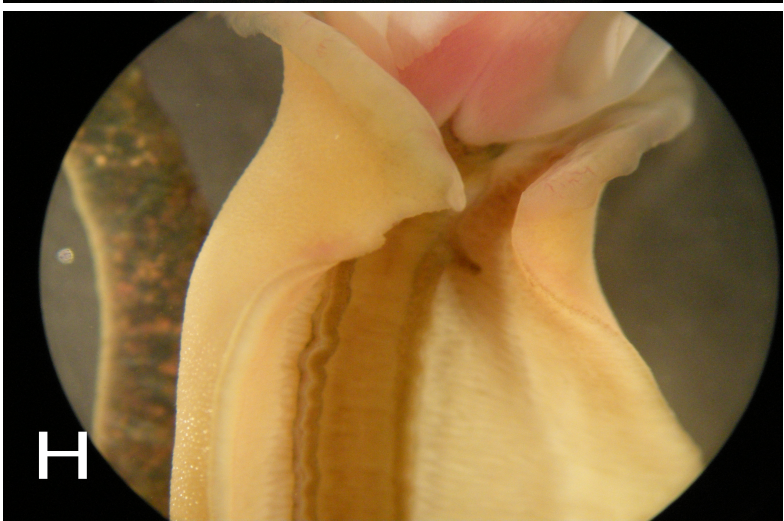

Supplement: Supplementary file 1 — Tubeworms recovered from Shrimp Hole at Von Damm, Mid-Cayman Rise with Remote Operated Vehicle Jason 2. Photographs: A, B In situ images of tubeworms on basalt C, D. Tubes of Escarpia and Lamellibrachia, showing anterior curved roots and higher magnification of tube opening, respectively, EF, Escarpia and GH, Lamellibrachia specimens extracted from tubes showing, branchial plumes (E-G) and trophosome (F, H). Photographs credit: Woods Hole Oceanographic Institution. (PDF 21694 kb) [file 40168_2018_411_MOESM1_ESM.pdf]

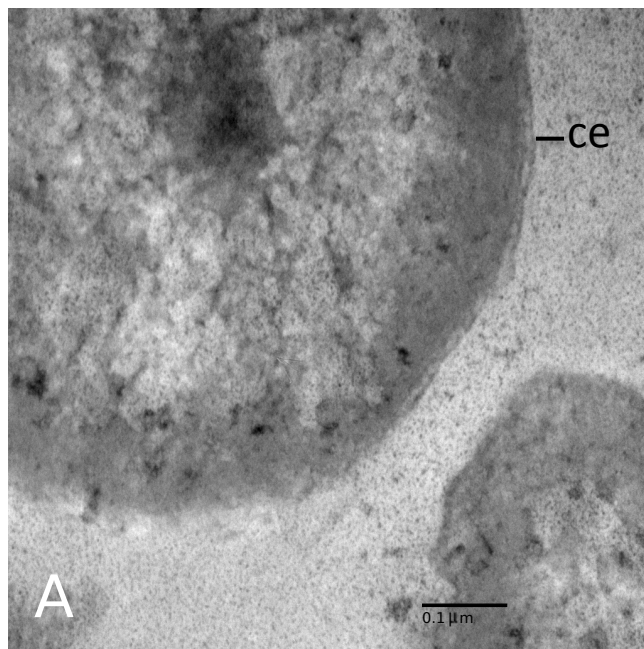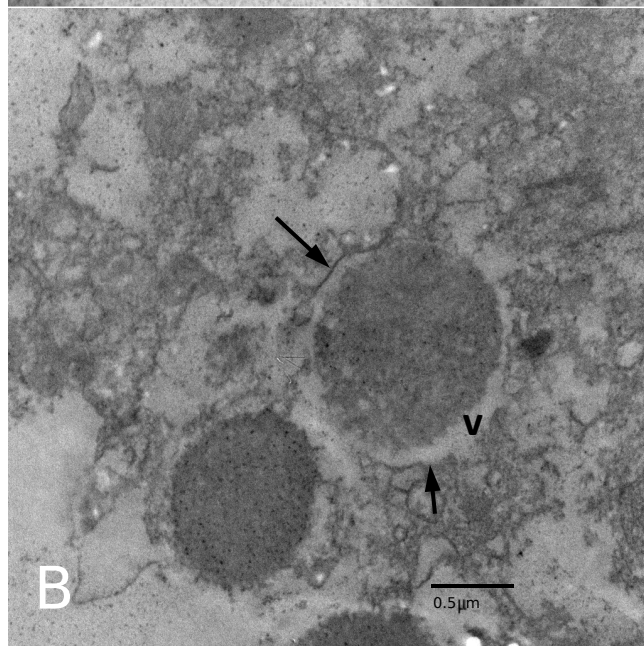

Supplement: Supplementary file 4 — Transmission electron micrographs of trophosome tissue of Mid-Cayman Rise vestimentiferans, Escarpia sp.(A) and Lamellibrachia sp.2 (B). showing coccoid endosymbionts with cell envelopes (ce) resembling those of Gram negative bacteria. The symbionts are typically surrounded by an additional membrane (arrows) suggesting they are contained within membrane-bound vacuoles (v) in host cells as in other vestimentiferans symbioses (PDF 1793 kb) [file 40168_2018_411_MOESM4_ESM.pdf]

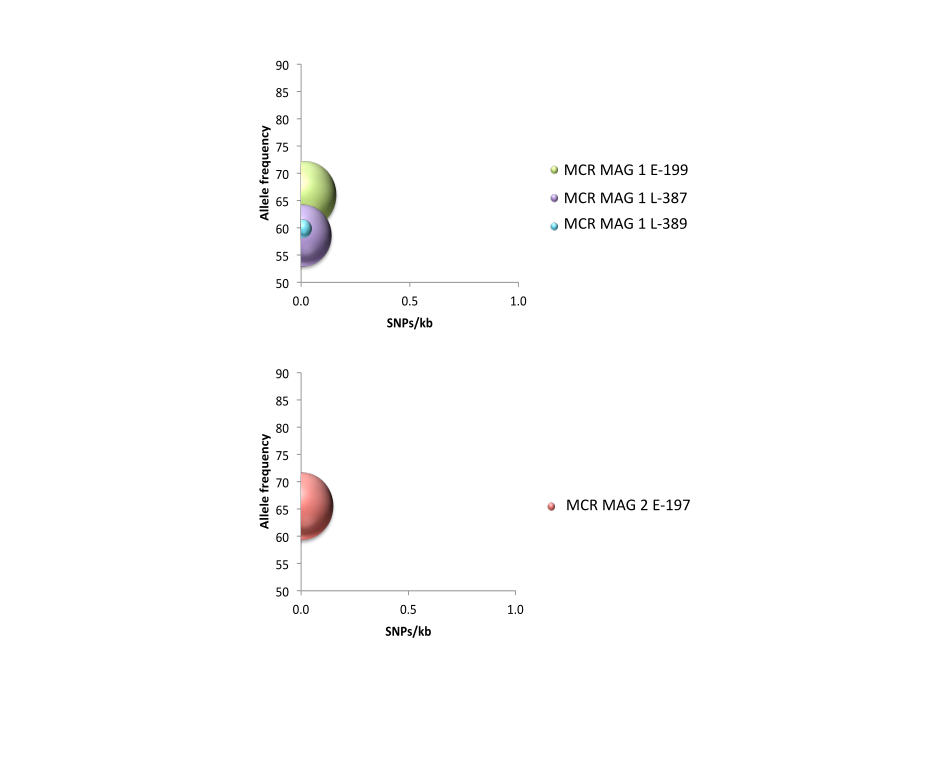

Supplement: Supplementary file 6 — Relationships between Single Nucleotide Variant (SNV) density/kb, SNV average allele frequency, and mean coverage for each MCR genomic bin in the four different tubeworm individuals (L and E indicates Lamellibrachia and Escarpia, respectively, with specimen number following). Size of bubble indicates coverage, with the largest bubble corresponding to a coverage of 245X (E-199), the smallest one to 18X (L- 389) and the intermediate ones to 177X (E-197) and 206X (L-387). (PNG 46 kb) [file 40168_2018_411_MOESM6_ESM.png]
